# Identification of Probucol as a candidate for combination therapy with Metformin for Type 2 diabetes

**DOI:** 10.1038/s41540-023-00275-8

**Published:** 2023-05-23

**Authors:** Ranjitha Guttapadu, Kalyani Korla, Safnaz UK, Vamseedhar Annam, Purnima Ashok, Nagasuma Chandra

**Affiliations:** 1 IISc Mathematics Initiative, Indian Institute of Science, Bengaluru, Karnataka 560012 India; 2 Department of Biochemistry, Indian Institute of Science, Bangalore, Karnataka 560012 India; 3 Department of Pharmacology, K.L.E. University’s College of Pharmacy, Bangalore, Karnataka 560010 India; 4 Department of Pathology, Rajarajeshwari Medical College and Hospital, Bangalore, Karnataka 560074 India; 5 Centre for Biosystems Science and Engineering, Indian Institute of Science, Bengaluru, Karnataka 560012 India

**Keywords:** Virtual drug screening, Endocrinology

## Abstract

Type 2 Diabetes (T2D) is often managed with metformin as the drug of choice. While it is effective overall, many patients progress to exhibit complications. Strategic drug combinations to tackle this problem would be useful. We constructed a genome-wide protein-protein interaction network capturing a global perspective of perturbations in diabetes by integrating T2D subjects’ transcriptomic data. We computed a ‘frequently perturbed subnetwork’ in T2D that captures common perturbations across tissue types and mapped the possible effects of Metformin onto it. We then identified a set of remaining T2D perturbations and potential drug targets among them, related to oxidative stress and hypercholesterolemia. We then identified Probucol as the potential co-drug for adjunct therapy with Metformin and evaluated the efficacy of the combination in a rat model of diabetes. We find Metformin-Probucol at 5:0.5 mg/kg effective in restoring near-normal serum glucose, lipid, and cholesterol levels.

## Introduction

Type 2 Diabetes (T2D), which affects more than 420 million people worldwide and makes up 90% of all known diabetes cases, refers to a collection of metabolic disorders characterized by high blood glucose levels and insulin resistance. WHO reports that the incidence has increased more than 3.5-fold in the past three decades, with lifestyle and genetic factors playing a significant role. By 2030, it is estimated to be the seventh leading cause of death^1^. The disease is multifactorial and cannot be attributed to a single gene or pathway, leading to a high level of heterogeneity among individuals. Despite this heterogeneity, T2D is primarily managed by metformin monotherapy, a biguanide that improves glycemia via AMP-activated protein kinase (AMPK)-dependent and AMPK-independent mechanisms in the liver and the gut^2^. Metformin (MT) is used as a first-line oral agent due to its safety and efficacy. However, due to the progressive loss of β cells with disease progression, a significant proportion of patients treated with Metformin need alternate therapy. Lesser recommended monotherapies include GLP1-RA, SGLT2i, DPP4i, TZD, AGi, and SU/GLN. The glycemic control algorithm under the Comprehensive Type 2 Diabetes Management algorithm suggests a dual therapy of Metformin with the drugs mentioned above or Basal Insulin, Colesevelam, and Bromocriptine QR when the Hemoglobin A1C (HbA1c) concentrations are at 7.5–9%^3^.

The need for effective combinations is even more pronounced when co-morbidities play a part or to alleviate the side effects of treatment. Identifying drug combinations that can effectively target different complications of diabetes would serve as more promising treatments that can translate into active use. Recent studies indicate that oxidative stress and hypercholesterolemia generated due to metabolic alterations are two main problems leading to complexity in T2D management. Oxidative stress and cholesterol levels are pivotal in developing diabetic complications such as nephropathy^4^, retinopathy^5^, cardiomyopathy^6^, and ketoacidosis^7^. Thus, the goal of treating T2D, in addition to maintaining blood glucose levels, must address oxidative stress and hypercholesterolemia.

In this work, we intend to identify candidate drugs that can be placed in combination with Metformin to target oxidative stress and hypercholesterolemia and reduce serum glucose levels. We first set out to identify perturbations in T2D patients and apply that knowledge to identify co-drugs to combine with Metformin. We leverage publicly available transcriptomes from T2D tissue samples, construct condition-specific networks, and interrogate them using sensitive network mining methods to identify the highest-ranked perturbations and strategic combinations. We identify Probucol as the co-drug of choice from the perturbations and experimentally validate the efficacy of the combination in vivo using diabetic rat models. Compared to individual drugs, we find the combination to be highly efficacious in lowering serum glucose, oxidative stress, and cholesterol levels.

## Results

**Fig. 1:**
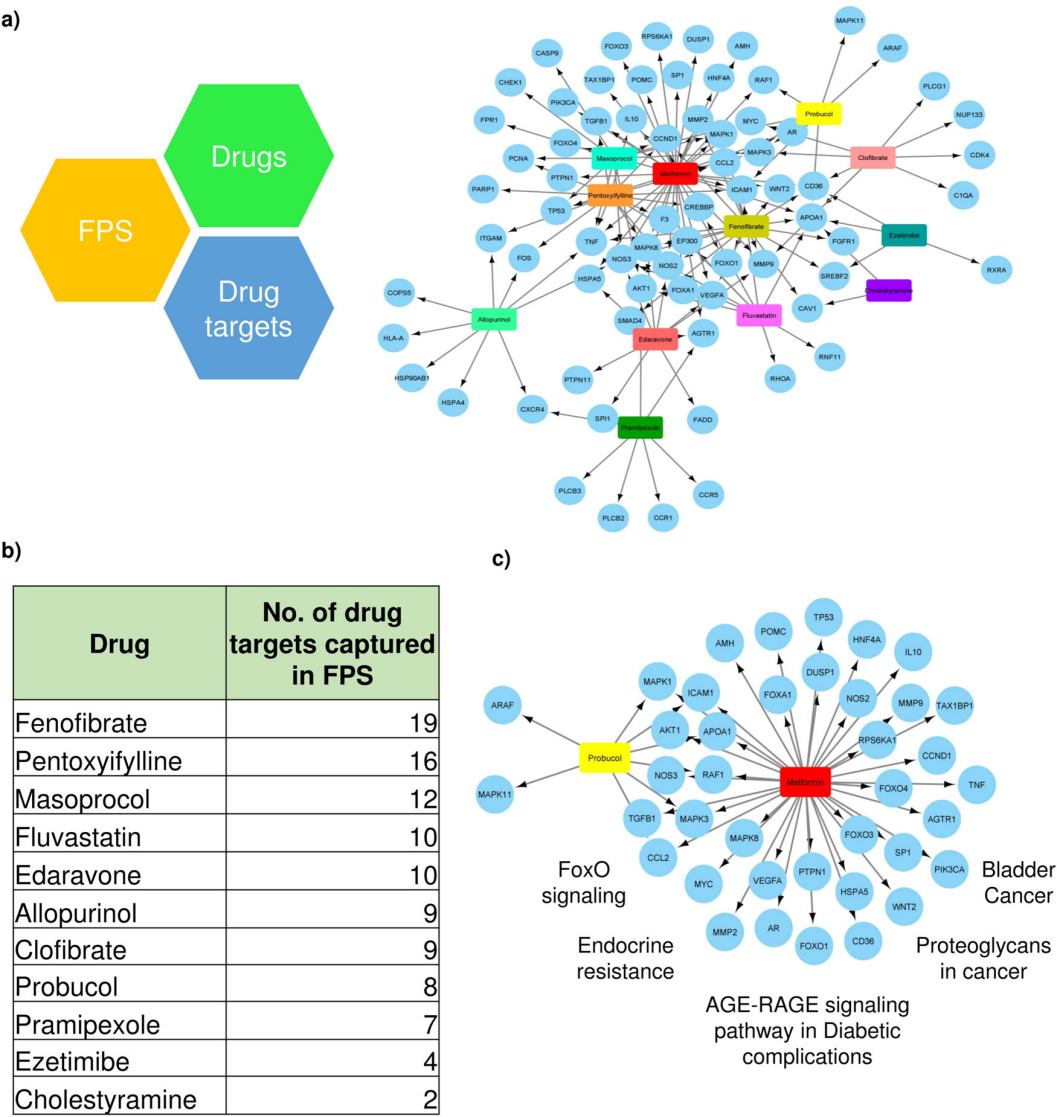
Network approach used to identify the potential co-drug candidate for Metformin. **a** Drug-target bipartite network of the shortlisted drugs and their targets in the FPS. **b** Drugs were ranked based on the number of known targets captured by the FPS and further filtered based on literature and mechanism of action. **c** Sub-bipartite network of Probucol, Metformin, and their targets. Probucol was shortlisted as the potential co-drug, and a sub-bipartite network of Probucol, Metformin, and their targets was generated from the parent bipartite network. Metformin is represented in red, Probucol in yellow, blue nodes represent target genes in the FPS, and the top 5 KEGG pathways (based on *p*-value (Fisher’s exact test)) in which the genes are enriched are indicated.

The broad workflow included: (a) analysis of publicly available human T2D transcriptomes from multiple tissues, (b) integrating these into an unbiased knowledge-based protein-protein interaction network, (c) network mining and identification of a frequently perturbed subnetwork (FPS), (d) identification of putative drug targets, (e) identification of a putative set of drugs associated with the targets and ranking them (Fig. 1) and (f) experimental testing of the effect of the selected drug combination.

### Heterogeneity in T2D patients

Publicly available transcriptome data for T2D individuals from four tissue types: pancreas, liver, skeletal muscle, and arterial tissue, were chosen for the study (as mentioned in the methods). Some known T2D genes were among the DEGs of the two pancreatic datasets (e.g., Solute Carrier Family 2 Member 2 (*SLC2A2*), Calcium Voltage-Gated Channel Subunit Alpha1 D (*CACNA1D*), which are known to play an essential role in T2D^8^, and 3-Hydroxy-3-Methylglutaryl-CoA Reductase (*HMGCR*), which is involved in cholesterol biosynthesis) but no common DEGs were observed across all the five datasets. The limited overlap could result from multiple factors, including the extent of genetic heterogeneity of different tissue types, the severity of disease, technical differences in data collection due to platform-based dependencies for individual datasets, and perhaps more importantly, variations in the initial causes of the disease itself. Heterogeneity in the individuals could translate into heterogeneity in the benefit experienced with metformin monotherapy underscoring the dire need for drug combinations.

However, the DEGs obtained in each dataset were enriched in pathways related to diabetes, such as alteration in glycolysis, pyruvate metabolism, cholesterol metabolism, Insulin/IGF pathway, pancreatic secretion, and MAPK signaling, all of which are well-known to be associated with T2D^9,10^. A list of all DEGS obtained is provided in Supplementary Data 1 (Sheet 1).

### Network meta-analysis to identify co-drug with Metformin in T2D patients

Next, we constructed condition-specific networks for each dataset using a sensitive network mining approach well established in our laboratory called ‘ResponseNet’ to identify perturbations in T2D across different tissue types. The top perturbed networks were obtained by mapping transcriptomic data onto a well-curated human protein-protein interaction network and ranking the nodes based on their activity via the shortest path analysis to capture genes representing variations in the studied condition. The mapping of transcriptomic data onto the knowledge-based protein-protein interaction network is done by contextualizing the network by overlaying the absolute value of log_2_(Fold Change (FC)) for each network gene from differential expression of genes analysis of the chosen datasets and using them as node weights. The activity of paths from the source node to the target nodes is calculated as the sum of edge weights along the shortest path, normalized by the path length. The approach is explained in the methods section. The lower the normalized path cost, the higher the activity. The networks were pooled to obtain a parent network representing perturbations in T2D across various tissues. The combined network consisted of 6829 nodes and 13,242 edges. From this, we extracted nodes present in at least 4 out of the 5 datasets representing the most frequently perturbed genes across tissues, forming a well-connected subnetwork (FPS). The FPS consisted of 225 nodes and 471 edges and was enriched in functional categories of AGE-RAGE signaling in diabetic complications, lipid signaling, and cholesterol metabolism, besides essential pathways such as chemokine signaling. Antioxidants, lipid-modifying, or anticholesterolemic agents, thus, form effective combinations with Metformin to target functional pathways reflected in the FPS.

To identify drug combinations with Metformin with a higher polypharmacological potential that can also tackle oxidative stress and hypercholesterolemia, we investigated if any of the nodes in the FPS are known drug targets of the 11 putative drug candidates shortlisted from DrugBank based on their antioxidant, lipid-modifying, and anticholesterolemic properties (Fig. 1a). The drugs were ranked based on the number of targets of each drug captured by the FPS (Fig. 1b).

37 metformin targets were present in FPS, indicating that the FPS functions well in representing core perturbations targeted by Metformin. Metformin’s combination with Fenofibrate is reported to be not beneficial^11^, while its combination with pentoxifylline was found to improve liver function in patients with non-alcoholic steatohepatitis^12^. The combination of Allopurinol and Metformin has been shown to act synergistically in Non-Alcoholic Fatty Liver Disease patients^13^. A combination of Metformin and Clofibrate has also been found to improve glucose levels^14^. Metformin is reported to increase fluvastatin’s hypolipidemic activities and decrease the excretion rate of Pramipexole^15^. The fact that FPS captures targets of many tested drug combinations provides proof of the principle of the effectiveness of our networks.

To find an effective drug combination, we filtered the drug list (Supplementary Data 1 (Sheet 2)) to obtain the top 3 drugs (Masoprocol, Edaravone, and Probucol (PB)) whose combinations with Metformin were unexplored. Masoprocol is used to treat actinic keratoses and is known to reduce glucose levels^16^, while Edaravone relieves neurological symptoms and delays the progression of ALS, and is implicated as a treatment strategy for diabetic neuropathy^17^. However, they do not directly tackle hypercholesterolemia. On the other hand, Probucol is an antioxidant that inhibits the oxidation of cholesterol. It is a drug indicated as an antioxidant, a lipid modifying agent, and an anticholesterolemic drug^15^. Since we aimed to address oxidative stress and hypercholesterolemia with our drug combination, Probucol was chosen as the target co-drug to explore its combinatorial effect with Metformin. Probucol is reported to be a potentially efficacious drug to treat T2D^18^ and related complications such as diabetic nephropathy^19^. However, its efficacy in combination with Metformin was unknown.

A bipartite network of drug targets for Probucol and Metformin with their targets from the FPS yielded a network of 41 nodes and 45 edges (Fig. 1c). Functional enrichment indicated top pathways: FoxO signaling, AGE-RAGE signaling in diabetic complications, and insulin resistance. The two drugs were also found to have six overlapping targets, including APOA1, RAF1, MAPK3, CCL2, ICAM1, and MAPK1, that play a role in pathways such as lipid and atherosclerosis and TNF signaling, a pathway known to play a part in insulin resistance^20^, further supporting our hypothesis of using the PB-MET combination to tackle oxidative stress and hypercholesterolemia along with serum glucose levels and insulin resistance in T2D.

## Experimental testing of the efficacy of the combination

### Acute toxicity analysis for drug combination

Acute toxicity studies for MET, PB, and the PB-MET combination were conducted per OECD guidelines 425, using female albino Wistar rats. The test group of animals was treated with MET, PB, or PB-MET through oral administration and compared with an untreated control group. The animals were continuously observed for the first 2 h and then after 24 h and 48 h. No change in the typical behavioral pattern of the animals, mortality, or signs and symptoms of toxicity was observed in any of the treatment regimes. The combination was observed safe up to a dose of 2000 mg/kg b.w.

### Anti-hyperglycemic and anti-hypercholesterolemic effects of drug combination

Different groups of rats (normal control- no treatment, diabetic control, diabetic treated (MET, PB, PB-MET)) were given the appropriate treatment as described in the methods section. At the end of the 28th day, their biochemical parameters were assessed to test the administered drugs’ anti-hyperglycemic, anti-hypercholesterolemic, and antioxidant effects. To quantify the anti-hyperglycemic impact, serum glucose, insulin, and HbA1c were measured. Lipid profiles, including High-Density Lipoprotein (HDL), Low-Density Lipoprotein (LDL), total cholesterol (TC), and triglyceride (TG), were estimated using serum samples to evaluate the anti-hypercholesterolemic effect. Serum nitric oxide and serum Thiobarbituric acid reactive substances (TBARS) were measured to test if there were any antioxidant effects. The study was repeated twice (Set-I and Set-II), using a similar grouping of animals with overlapping ranges of drug doses.

**Fig. 2:**
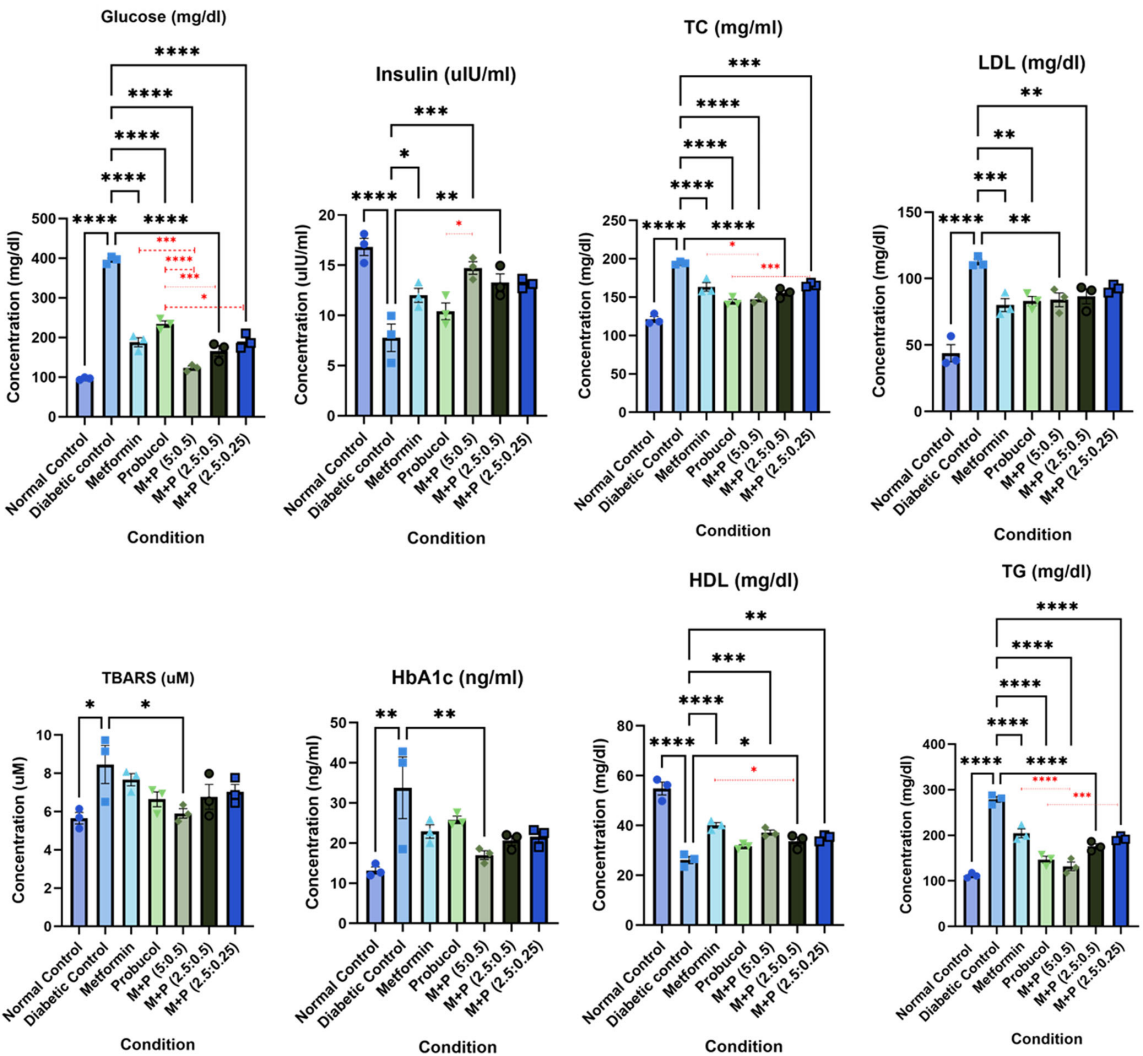
Graphical representation of the biochemical parameters measured in Set II. Statistical significance was calculated using ANOVA followed by Dunnett’s test (*p-value < 0.05, **p-value < 0.01, ***p-value < 0.001, ****p-value < 0.0001). Mean with S.E.M are plotted. M + P (5:0.5) shows concentrations closest to the normal healthy control for all parameters and hence is chosen as the ideal dosage concentration for the drug combination. See also Supplementary Fig. 1. The significance bars in black indicate statistical significance calculated against normal/diabetic control, while the ones in red indicate statistical significance calculated against metformin/probucol monotherapies.

In both sets, the induction of diabetes was first established in the diabetic control and test groups. Results from Set II are shown here, while results from Set I are included as a supplementary file. All *p*-values reported for the biochemical parameters were calculated using ANOVA followed by Dunnett’s test. A significant increase in fasting serum glucose (395.4 ± 5.37 mg/dl; adjusted *p*-value < 0.0001, 95% CI [262.7, 335.2], df = 14, *F* = 123.3), serum HbA1c levels (33.79 ± 7.68 ng/ml; adjusted *p*-value = 0.0018, 95% CI [7.802, 33.40], df = 14, *F* = 4.46), and a decrease in serum insulin (7.78 ± 1.36 uIU/ml; adjusted *p*-value < 0.0001, 95% CI [−12.53, −5.564], df = 14, *F* = 12.02) was detected in the untreated diabetic rats compared to normal rats (serum glucose - 96.49 ± 1.74 mg/dl, HbA1c- 13.19 ± 0.88 ng/ml, serum insulin- 16.82 ± 0.85 uIU/ml) (Table 1, Fig. 2; Supplementary Table 1, Supplementary Fig. 1). MET alone, as expected, reversed these trends and reduced serum glucose to 188.0 ± 11.41 mg/dl (adjusted *p*-value < 0.0001, 95% CI [171.2, 243.7], df = 14, *F* = 123.3), HbA1C to 22.88 ± 1.66 ng/ml (adjusted *p*-value = 0.109, 95% CI [−1.891,23.71], df = 14, *F* = 4.46), while increasing insulin levels to 12.01 ± 0.70 uIU/ml; adjusted *p*-value = 0.0154, 95% CI [−7.716, −0.748], df = 14, *F* = 12.02) compared to the diabetic control. PB alone also showed an anti-hyperglycemic effect, albeit less potent and less efficacious than MET, with serum glucose reducing to 233.8 ± 7.96 mg/dl (adjusted *p*-value < 0.0001, 95% CI [125.4, 297.9], df = 14, *F* = 123.3), HbA1C to 25.79 ± 0.92 ng/ml (adjusted *p*-value = 0.3203, 95% CI [−4.795, 20.80], df = 14, *F* = 4.46), and insulin levels increasing to 10.41 ± 0.84 uIU/ml; adjusted *p*-value = 0.1763, 95% CI [−6.12, 0.85], df = 14, F = 12.02) compared to the diabetic control. The PB-MET combination, however, showed a more significant decrease in serum glucose (e.g., M + P (5:0.5 mg/kg)- 123.2 ± 4.70 mg/dl; adjusted *p*-value < 0.0001, 95% CI [236.0, 308.6], df = 14, *F* = 123.3) and HbA1c (e.g., M + P (5:0.5 mg/kg)- 17.02 ± 1.07 ng/ml; adjusted *p*-value = 0.0090, 95% CI [3.97, 29.57], df = 14, *F* = 4.46) and a corresponding increase in serum insulin levels (e.g., M + P (5:0.5 mg/kg)- 14.73 ± 0.63 uIU/ml; adjusted *p*-value = 0.0003, 95% CI [−10.44,−3.473], df = 14, F = 12.02) compared to the diabetic control group, a reduction more substantial than either MET or PB monotherapy. At the end of 28 days of treatment, serum insulin, and HbA1c levels were brought close to the levels of the normal control group (Table 1, Fig. 2; Supplementary Table 1, Supplementary Fig. 1).

**Table 1:** Biochemical parameters studied in Set II.

Condition	Lipid profile (mg/dl)				Serum glucose (mg/dl)	Serum HbA1c (ng/ml)	Serum Insulin (uIU/ml)	Serum TBARS (uM)
	Total cholesterol	Triglycerides	HDL-C	LDL-C				
Normal Control	121.1 ± 3.83	111.9 ± 3.29	54.83 ± 2.57	43.90 ± 6.42	96.49 ± 1.74	13.19 ± 0.88	16.82 ± 0.85	5.65 ± 0.30
Diabetic control	194.4 ± 1.21^****a^	278.9 ± 6.30^****a^	26.14 ± 1.45^***a^	112.5 ± 2.16^****a^	395.4 ± 5.37^***a^	33.79 ± 7.68^**a^	7.78 ± 1.36^****a^	8.46 ± 0.99^*a^
Metformin (5)	163.5 ± 5.67^****b^	204.6 ± 9.63^****b^	39.43 ± 0.60^***b^	80.04 ± 4.90^***b^	188.0 ± 11.41^***b^	22.88 ± 1.66	12.01 ± 0.70^*b^	7.67 ± 0.32
Probucol (5)	144.1 ± 3.20^****b^	146.6 ± 7.49^****b^	31.62 ± 0.64^*b^	83.12 ± 3.44^**b^	233.8 ± 7.96^***b^	25.79 ± 0.92	10.41 ± 0.84	6.64 ± 0.39
M + P (5: 0.5)	147.3 ± 2.54^****b *c^	132.2 ± 9.25^****b ****c^	36.84 ± 1.27^***b^	84.00 ± 5.24^**b^	123.2 ± 4.70^***b ***c ****d^	17.02 ± 1.07^**b^	14.73 ± 0.63^***b *d^	5.91 ± 0.25^*b^
M + P (2.5: 0.5)	155.5 ± 3.70^****b^	175.7 ± 6.04^****b^	34.68 ± 0.69^**b *c^	86.66 ± 5.63^**b^	166.1 ± 13.43^***b***d^	20.65 ± 1.16^*b^	13.27 ± 0.88^**b^	6.77 ± 0.65
M + P (2.5 + 0.25)	166.1 ± 1.54^***b ***d^	193.0 ± 3.09^****b ***d^	35.06 ± 0.73^**b^	92.47 ± 1.70^*b^	190.1 ± 10.71^***b *d^	21.61 ± 1.30	13.17 ± 0.27^**b^	7.03 ± 0.39

Values are expressed as Mean ± S.E.M, ANOVA followed by Dunnett’s multiple comparison test. ^a^compared to the normal group, ^b^compared to the diabetic group, ^c^compared to the metformin monotherapy group, ^d^compared to the probucol monotherapy; *p-value < 0.05, **p-value < 0.01, ***p-value < 0.001, ****p-value < 0.0001. See also Supplementary Table 1.

The serum triglyceride, cholesterol, and LDL levels of untreated diabetic rats were significantly higher (278.9 ± 6.30 mg/dl (adjusted *p*-value < 0.0001, 95% CI [138.6, 195.3], df = 14, *F* = 65.44), 194.4 ± 1.21 mg/dl (adjusted *p*-value < 0.0001, 95% CI [59.29, 87.29], df = 14, *F* = 44.25), 112.5 ± 2.16 mg/dl (adjusted *p*-value < 0.0001, 95% CI [49.92, 87.27], df = 14, *F* = 20.37) respectively) than those in healthy rats, and HDL level was significantly lower (26.14 ± 1.45 mg/dl; adjusted *p*-value < 0.0001, 95% CI [−34.63, −22.77], df = 14, *F* = 39.36) than the normal control (Triglycerides − 111.9 ± 3.29 mg/dl, TC- 121.1 ± 3.82 mg/dl, LDL- 43.90 ± 6.42 mg/dl). The administration of MET and PB alone and in combination significantly decreased triglyceride (e.g., M + P (5:0.5 mg/kg)− 132.2 ± 9.25 mg/dl; adjusted *p*-value < 0.0001, 95% CI [118.3, 175], df =14, F = 65.44), total cholesterol (e.g., M + P (5:0.5 mg/kg)− 147.3 ± 2.54 mg/dl; adjusted *p*-value < 0.0001, 95% CI [33.12, 61.13], df = 14, *F* = 44.25), and LDL levels (e.g., M + P (5:0.5 mg/kg)- 84.00 ± 5.24 mg/dl; adjusted *p*-value = 0.0028, 95% CI [9.82,47.17], df = 14, *F* = 20.37) and increased HDL levels (e.g., M + P (5:0.5 mg/kg)- 36.84 ± 1.27 mg/dl; adjusted *p*-value = 0.0005, 95% CI [−16.96, −5.10], df = 14, *F* = 39.36) during the experimental period when compared to the diabetic rats. (Table 1, Fig. 2; Supplementary Table 1, Supplementary Fig. 1). All *p*-values were calculated using ANOVA followed by Dunnett’s test.

The PB-MET combination at the dosage M + P (5:0.5) seems to behave synergistically, producing a significant hypoglycemic effect in terms of reducing the serum glucose and HbA1c lower than the response generated by the individual drugs alone while also increasing the serum insulin to a larger extent than metformin or probucol treatment alone. Synergy was also observed in triglyceride reduction. The combination treatment was found to fare better than individual monotherapies, thus showing an anti-hyperlipidemic effect in the test group, demonstrating its efficacy in reducing diabetes-induced hyperlipidemia in rats.

### Antioxidant effect

In Set II, TBARS was used as an oxidative stress indicator. Lower levels of TBARS indicate a lower level of oxidative stress and, thus, a better antioxidant effect. As seen in Table 1, the levels of TBARS in M + P (5: 0.5) (5.91 ± 0.25 uM) were close to normal (5.65 ± 0.3 uM) and were highest in the diabetic control group (8.46 ± 0.99 uM; adjusted *p*-value = 0.01, 95% CI [0.63, 4.99], df = 14, *F* = 3.38 when compared to the normal control). The drug combination seems to act synergistically in reducing the antioxidant effect as well, where M + P (5: 0.5) produced 5.91 ± 0.25 uM TBARS, a concentration less than (adjusted *p*-value = 0.02, 95% CI [0.38, 4,73], df = 14, *F* = 3.38 when compared to the diabetic control) that produced by metformin (7.67 ± 0.32 uM; adjusted *p*-value = 0.78, 95% CI [−1.38, 2.98], df = 14, *F* = 3.38 when compared to the diabetic control) or probucol monotherapy (6.64 ± 0.39 uM; adjusted *p*-value = 0.12, 95% CI [0.36, 4.00], df = 14, *F* = 3.38 when compared to the diabetic control) (Table 1, Fig. 2). All *p*-values were calculated using ANOVA followed by Dunnett’s test.

The M + P (5:0.5) dose provided the best results by reverting T2D to almost similar concentrations of parameters as the healthy controls (Table 1, Fig. 2; Supplementary Table 1, Supplementary Fig. 1). Individual rat data for all biochemical analyses can be accessed in Supplementary Data 2.

### Histopathology

**Fig. 3:**
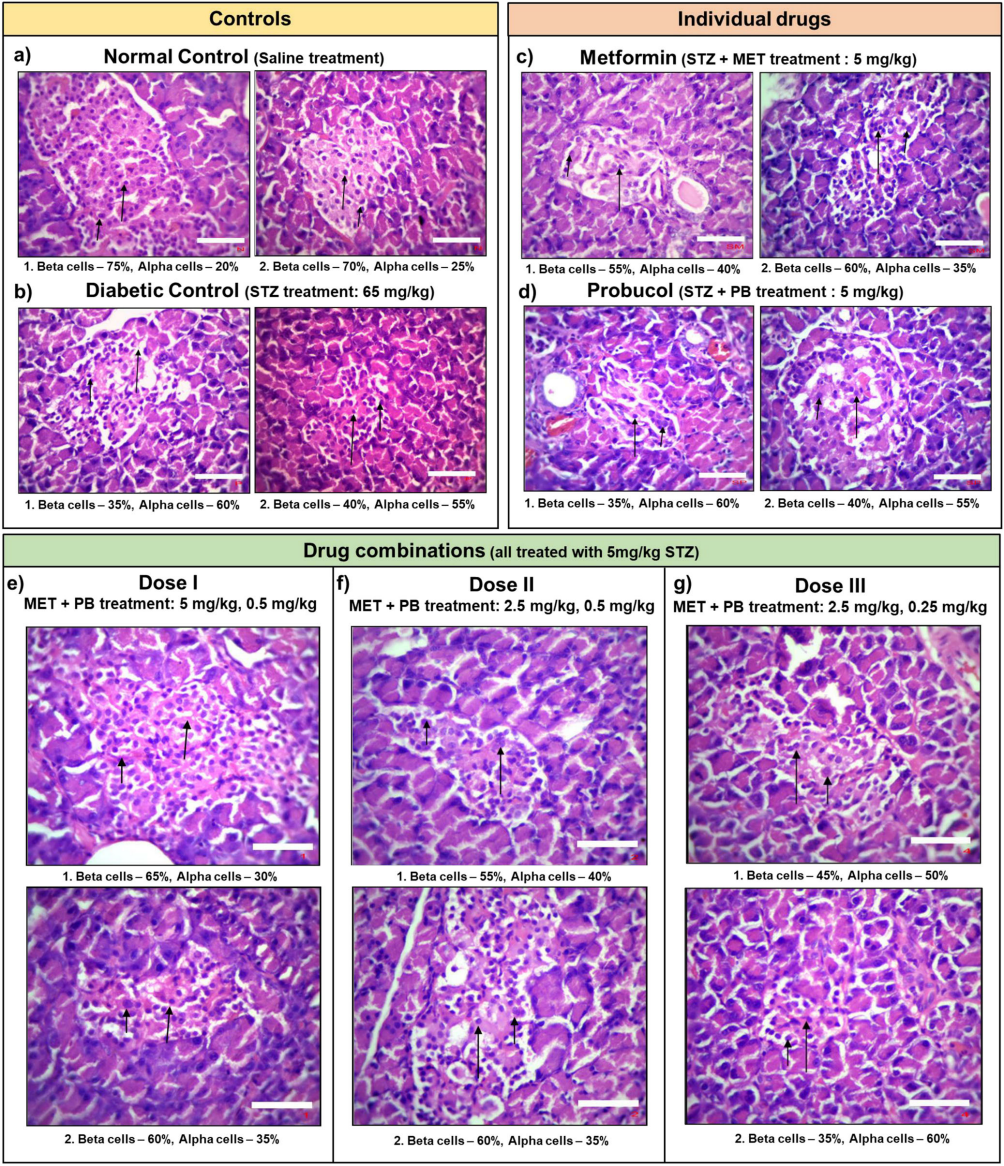
Histopathological analysis of pancreatic tissue. Tissue was obtained from NAD-STZ induced diabetic model (400x; scale bars = 25 µm). All analyses were performed in duplicates (1, 2). The sections studied showed pancreatic lobules separated by connective tissue septa. The center of islet cells consists of Beta-cells (Long-arrow), while the periphery comprises large Alpha-cells (Short-arrow) with intervening vascular spaces. The pancreatic lobules consist largely of the exocrine acini and their intralobular ducts. Most of the lobules show small, round, light-staining islets of Langerhans. **a**, **b** Controls – Normal and Diabetic. Some of the beta cells show degenerative changes in the positive control. **c**, **d** Individual drug treatment - Metformin and Probucol treated. **e** Drug combination M + P (5:0.5). **f** M + P (2.5:0.5). **g** M + P (2.5:0.25). Dose I [M + P (5:0.5)] restored the condition of cells closest to that of the normal tissue conditions. See also Supplementary Fig. 2.

To further test if there were morphological changes associated with drug treatment, hematoxylin-eosin staining was performed on the rats’ pancreatic tissue sections. Results of Set I are shown in Supplementary Fig. 2. Drug combinations at decreasing doses and controls from Set II were analyzed in duplicates. Healthy non-diabetic tissue sections studied showed pancreatic lobules separated by connective tissue septa. One of the replicates’ center of islet cells consisted of 75% Beta-cells, while the periphery consisted of 20% large Alpha-cells (Fig. 3a. 1). The second replicate consisted of 70% Beta-cells, while the periphery consisted of 25% large Alpha-cells (Fig. 3a. 2). The center of islet cells of the diabetic control showed a quantitative decrease in small Beta-cells (Replicate 1- 35%, Replicate 2 - 40% compared to normal control), while the periphery comprised large Alpha-cells (Replicate 1 - 60%, Replicate 2 - 55%) (Fig. 3b. 1, 2). Some of the beta cells showed degenerative changes. Tissue treated with Metformin alone showed lobules consisting mainly of the exocrine acini and their intralobular ducts. Most of the lobules showed small, round, light-staining islets of Langerhans. The center of islet cells consisted of aggregates of small Beta-cells (Replicate 1 - 55%, Replicate 2- 60%), while the periphery consisted of large Alpha-cells (Replicate 1 - 40%, Replicate 2- 35%) (Fig. 3c. 1, 2). Similar morphology was observed for tissues from rats treated with Probucol alone with 35% and 40% Beta-cells in Replicate 1 and Replicate 2, respectively and 60% and 55% of Alpha-cells in Replicate 1 and Replicate 2, respectively (Fig. 3d. 1, 2).

Dose I treatment (M + P [5:0.5]) led to a quantitative increase in Beta-cells (Replicate 1 - 65%, Replicate 2- 60%) and a decrease in Alpha-cells (Replicate 1 - 30%, Replicate 2- 35%) (Fig. 3e. 1, 2). Tissue from Dose II (M + P [2.5:0.5]) treated rats showed 55% Beta-cells, 40% Alpha-cells, and 60% Beta-cells and 35% Alpha-cells in Replicate 1 and Replicate 2, respectively (Fig. 3f. 1, 2), while Dose III (M + P [2.5:0.25]) treated rats showed 45% Beta-cells, 50% Alpha-cells and 35% Beta-cells and 60% Alpha-cells in Replicate 1 and Replicate 2 respectively (Fig. 3g. 1, 2). The Dose I, with a combination of 5 mg/kg of Metformin and 0.5 mg/kg of Probucol, showed a reversal of tissue morphology to a state similar to that of healthy tissues in both Set I and Set II, similar to the results obtained from the biochemical analyses.

These results show that the PB-MET drug combination helps preserve beta-cell function while also tackling the complications of insulin deficiency and resistance, as suggested by the network analysis.

## Discussion

T2D, as discussed, is caused by multiple alterations at the cellular and molecular levels, with several genes involved globally, leading to multifactorial pathogenesis^21^. It is well understood that biological systems such as this exhibit complex connections among individual players of the system and are often tightly coordinated and regulated^22,23^. It is not surprising, therefore, that an alteration in a single gene or protein often does not result in disease. A disease can be depicted as a pathophysiological state perturbing the underlying complex interdependent molecular network. In the background of this fundamental complexity, a systems approach becomes critical to studying variations. Network formulations offer an elegant method to study perturbations in complex systems and provide insight into the mechanisms of perturbations.

In this study, we propose a systems biology approach to rationally identify drugs that can be combined for efficient disease management and related complications of T2D. We identified genes that showed variation in their gene expression pattern in five transcriptomic datasets across different tissue types. The results indicated that none of the DEGs were common to all the datasets, which suggests that heterogeneity in the genotype space is widespread. However, a similar phenotype with diabetes-related pathways was identified, implying that while each study may not report the same DEGs, they may highlight DEGs belonging to common functional categories. The network-based approach, combined with literature knowledge, led to the selection of Probucol as a co-drug for metformin therapy. Probucol is an antioxidant that effectively combats oxidative stress^24^ and reduces blood cholesterol. As mentioned, a few studies have shown the potential of using Probucol to treat diabetes, such as preserving pancreatic function and modulating the development of diabetic cardiomyopathy^25^ and nephropathy^26^. Despite these studies, a combination of Metformin and Probucol has not been studied. As a preliminary study, we have shown experimentally that this combination performs better than the individual drugs on diabetic rats by monitoring parameters such as serum glucose, cholesterol, and oxidative stress.

We used different dosages and found that a combination of MET + PB (5 mg/kg, 0.5 mg/kg) performed the best among others. A lower dosage of Probucol wasn’t effective for obvious reasons, whereas a higher dosage also showed a decline in efficacy, which could be attributed to its HDL-lowering effect, thus indicating that overdosage may be detrimental.

Further, drug synergism was also established experimentally, where we demonstrated that Metformin, in combination with Probucol, has significantly enhanced anti-hyperglycemic efficacy compared to their individual monotherapies. Diabetic rats with significant (adjusted *p*-value < 0.001(ANOVA + Dunnett’s test)) elevation in serum glucose and reduced serum insulin values were treated with individual drugs and varying doses of metformin and probucol. Treatment with the combination therapy improved hyperglycemia by substantially reducing glucose compared to individual drug treatments. While metformin and probucol monotherapy resulted in a serum glucose level of 188.0 ± 11.41 mg/dl and 233.8 ± 7.96 mg/dl, respectively, when compared to the 395.4 ± 5.37 mg/dl serum glucose level of diabetic mice, indicating a 52.4% and 40.8% reduction respectively, combinatorial treatment with the two drugs provided further lowering of serum glucose. M + P (5:5), M + P (5:1), and M + P (5:0.5) resulted in serum glucose levels of 135.9 ± 4.18 mg/dl, 104.3 ± 1.18 mg/dl, 123.2 ± 4.70 mg/dl respectively. The glucose concentrations, however, increased upon further lowering of the drug dosages, leading us to identify the drug dosages at M + P 5:0.5 mg/kg to be an ideal synergistic drug combination that provided a more favorable response when combined than through monotherapies. M + P (5:0.5) resulted in a 68.8% reduction of glucose when compared to diabetic rats (adjusted *p*-value < 0.0001), while a 34.4% (adjusted *p*-value = 0.0007) and 47.3% reduction (adjusted *p*-value < 0.0001) (all p-values calculated using ANOVA followed by the Dunnett’s test) compared to metformin and probucol monotherapies.

Synergy was also observed in lowering the triglyceride concentration where M + P 5:0.5 mg/kg treatment resulted in a 132.2 ± 9.25 mg/dl of triglycerides compared to a lower reduction using monotherapies with metformin alone at 5 mg/kg and probucol alone at 5 mg/kg leading to 204.6 ± 9.63 mg/dl and 146.6 ± 7.49 mg/dl of triglycerides respectively. This study also highlighted that both Metformin and Probucol, in combination, improved oxidative stress as reflected by a reduction in serum nitric oxide level and level of TBARS. M + P (5:0.5) resulted in a 5.91 ± 0.25 uM release of TBARS, a concentration less than that produced by metformin (7.67 ± 0.32 uM) or probucol monotherapies (6.64 ± 0.39 uM).

Although the combination (MET + PB) seems advantageous, validation in a larger cohort of animals is required to determine the best dose precisely. Currently, statins are prescribed along with Metformin for tackling cardiovascular complications in T2D patients but have been linked with increased risk of T2D^27^. Probucol seems promising for lowering LDL and coronary artery disease risk, especially in familial hypercholesterolemia and is a drug in active use in Japan^28,29^ and also a drug under phase II clinical trials for Alzheimer’s disease in Australia^30^. However, there are reports which suggest that due to possible QT prolongation^31,32^, it has been recalled from markets in some other countries^28,29^. In contrast, recent evidence has shown that probucol may be more beneficial than harmful, showing positive effects in high-risk patients with certain cardiovascular ailments^29^. Nevertheless, our data serves as a proof-of-concept of the use of probucol or a safer substitute as part of a strategic combination of drugs to lower glucose and lipid levels, which may have value in better management of diabetes and its complications.

## Methods

### Transcriptome datasets and DEG computation

NCBI’s Gene Expression Omnibus (GEO) was used to identify five transcriptomic datasets (GSE18732, GSE13760, GSE20966, GSE23343, and GSE25724) of the condition and matched controls from arterial tissue, pancreas, skeletal muscle, and liver samples. The raw microarray data were normalized using the Robust Multi-array Average (RMA) method, and the Limma package in R 3.4.1^33^ was used to identify significantly differentially expressed genes (DEGs) in T2D compared to their healthy controls (FC > 1.5 and *p*-value < 0.05 (calculated by the Empirical Bayes method)).

### Construction of condition-specific networks

An in-house network mining approach called ‘ResponseNet’ was used to identify the most perturbed gene interactions and pathways in T2D. Two inputs: (a) a knowledge-based protein-protein interaction network previously curated in the laboratory and (b) transcriptome data from selected datasets were used to construct diabetes-specific networks. The knowledge-based human protein-protein interaction (hPPIn) network used in the study is a directional network developed by curating protein-protein interactions from publicly available databases such as Kyoto Encyclopedia of Genes and Genomes (KEGG), OmniPath, SignaLink, TRRUST, RegNetwork, Harmonizome and Human Transcriptional Regulation Interaction Database (HTRI). Each node of the network represents a gene, and an interaction between two genes is represented as an edge. In order to make this network condition-specific (T2D specific), transcriptomic data, in particular, the absolute value of log_2_FC calculated between T2D vs. control of each dataset, were used as node weights (NW) (mapping of transcriptomic data onto the hPPIn). FC for a given gene *i* in T2D (D) condition with respect to control (C) condition was computed as:

1 $$F{C}_{i}=S{{I}_{i}}_{D}/S{{I}_{i}}_{C}$$

Where FC_i_ is the fold change of given gene *i*, SI_i(D)_ and SI_i(C)_ represent the normalized signal intensities of gene *i* in diabetic and control samples, respectively. Fold changes capture variations occurring in the condition being studied compared to a control, and thus the use of fold changes as node weights contextualizes the network to a condition-specific one. A T2D-specific network was thus generated for each tissue-specific dataset under study. Further, the edge weight (EW_ij_) for an edge between gene i and j was computed as:

2 $$E{W}_{{ij}}=\frac{1}{\sqrt{N{W}_{i}* N{W}_{j}}}$$

Where NW_*i*_ and NW_*j*_ are the node weights of gene *i* and *j*, respectively. EW_ij_ was used to compute all-vs-all shortest paths for all the genes in the network based on Dijkstra’s algorithm as described in Sambarey et al. ^34^ and Sambaturu et al. ^35^. In brief, Dijkstra’s algorithm was iteratively run from each node of the network to all other nodes; thus, finding all pairs shortest paths (all vs. all). The path cost of each shortest path was calculated as the sum of all edge weights from its source node to the target node along its shortest path. Dijkstra’s algorithm was chosen for finding all pair shortest paths as the time complexity would be O(VE Log V) which can go to (V3 Log V) in the worst case when running it for every vertex.

The shortest paths obtained are then ranked based on their activity which is described as the path cost normalized by their path length, which is equal to the number of edges between the source node and the target node. The top 0.01% of the ranked paths are considered the top perturbed paths or the top perturbed network (TopNet). A general version of the code can be accessed from https://github.com/NarmadaSambaturu/PathExt.

### Construction of a frequently perturbed subnetwork (FPS): Meta-network analysis

**Fig. 4:**
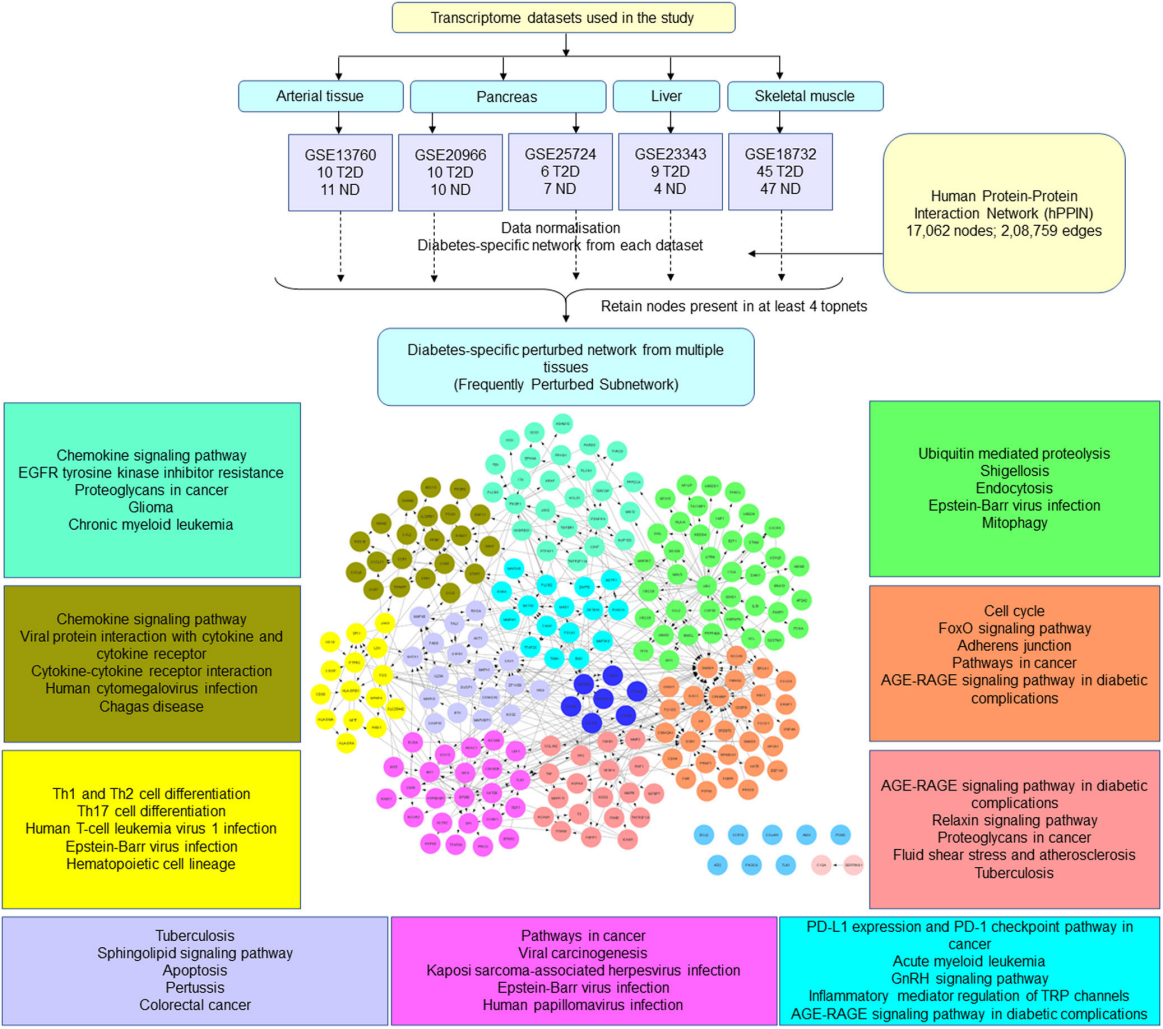
Overview of workflow to obtain the Frequently Perturbed Subnetwork (FPS). Transcriptomic datasets were obtained from the NCBI GEO database. T2D-specific response networks (TopNet) were generated from each dataset using a master human protein-protein interaction network (hPPIn). The topnets from all five datasets were combined, and the nodes which appeared in at least four topnets were retained to obtain a T2D-specific frequently perturbed subnetwork across multiple tissues. Functional clusters of the FPS are marked in different colors, and the top 5 most significantly enriched KEGG pathways are shown in boxes with the same colors. AGE-RAGE signaling in diabetic complications, an oxidative stress-induced pathway, seemed to be the most perturbed pathway captured by the FPS.

A T2D-specific network was generated for each dataset, and the top 0.01% paths from each of these networks were merged to obtain a combined network which was further filtered to retain nodes present in at least four of the five T2D networks. The resulting core perturbed network was termed the ‘Frequently Perturbed Subnetwork’ corresponding to perturbations in T2D across different tissue types. The Cytoscape plugin ReactomeFIPlugIn was used to cluster and analyze the corresponding functional pathways^36^. An overview of the workflow used to obtain the FPS is shown in Fig. 4.

### Identification of co-targets to complement the action of Metformin

To identify potential co-drugs for Metformin capable of addressing T2D complications of oxidative stress and hypercholesterolemia, drugs that are antioxidants (DBCAT000368), lipid-modifying (DBCAT002168), or anti-hypercholesterolemic (DBCAT000390) in their action were identified from the DrugBank database^15^ and analyzed for their impact on the FPS. A list of 11 such approved drugs, viz. Allopurinol, Cholestyramine, Clofibrate, Edaravone, Ezetimibe, Fenofibrate, Fluvastatin, Masoprocol, Pentoxifylline, Pramipexole, and Probucol were shortlisted based on their description. The targets for these drugs were compiled from the STITCH database^37^.

### Bipartite networks and choosing the co-drug

The perturbed pathways in a bipartite subnetwork with Metformin, the co-drug, and their targets captured in the FPS were analyzed using functional enrichment. All networks were visualized using Cytoscape 3.8.2. A directed drug-target interaction network was constructed using the 11 shortlisted drugs, Metformin, and their known targets present in the FPS. The drugs were ranked based on the number of known targets in the FPS. The list was further filtered based on literature mining, retaining only drugs previously not studied in combination with Metformin. Finally, one drug, probucol, was chosen as a potential co-drug for Metformin, as it is associated with oxidative stress and hypercholesterolemia. This approach helps identify drug combinations that target the most frequently perturbed genes across various tissue types in T2D. Functional enrichment for various gene sets and subnetworks was performed using the EnrichR server^38^ and the ReactomeFIPlugIn in Cytoscape. Pathways with a *p*-value < 0.05 (Fisher’s exact test) were considered significantly perturbed.

### In vivo studies

#### Drugs

Streptozotocin (S0130) was procured from Sigma Aldrich (St. Louis, MO, USA), Probucol (QA-2660), and Metformin (ST-9194) was purchased from Combi-Blocks, Inc. (San Diego, CA, USA).

#### Animals

Healthy Wistar albino rats weighing 150–180 g and 180–230 g were selected for acute oral toxicity study and anti-diabetic activity, respectively. The animals were procured from the Drug control department, Bengaluru, India, and housed in polypropylene cages maintained under standard hygienic laboratory conditions at a temperature of 23 ± 2 °C, relative humidity of 55 ± 5%, and 12 h light and dark cycles with free access ad libitum to a standard pellet diet and water. The experimental protocol was approved by KLE University’s College of Pharmacy, Bengaluru’s Institutional Animal Ethics Committee (Ref. no: 01/PA/2016).

#### Acute toxicity studies

Acute oral toxicity study for the drug combination was conducted per OECD guidelines 425 (“Test No. 425: Acute Oral Toxicity: Up-and-Down Procedure” 2008) using female Wistar albino rats weighing 150–180 g. Each animal was administered the drug combination orally at various doses up to 2000 mg/kg b.w. The animals were constantly observed for the first 2 h and up to 48 h for mortality or behavioral changes.

### Evaluation of anti-diabetic activity

#### Induction of T2D

Streptozotocin (STZ) was dissolved in 0.1 M cold citrate buffer (pH 4.5), and NAD was dissolved in normal physiological saline (0.9%). T2D was induced in rats under overnight fasting by administering NAD (110 mg/kg, i.p) 15 min before STZ (65 mg/kg, i.p). Induction of T2D was verified after 72 h, and the animals with blood glucose levels higher than 200 mg/dL were chosen for the study.

#### Experimental design

**Fig. 5:**
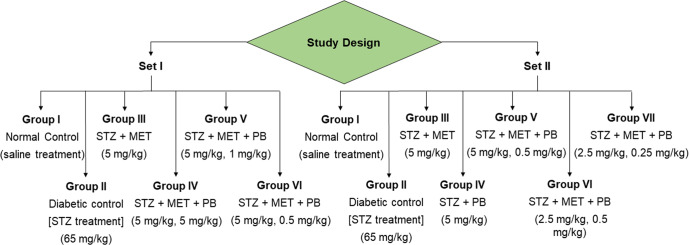
Experimental design. The experiments were split into two sets with overlapping ranges of drug doses. Animals in Group I and Group II served as the normal and diabetic control, respectively, in both studies. Group III of Set I, II served as metformin monotherapy control, while Group IV of Set II served as the probucol monotherapy control.

Wistar albino rats of either sex were randomly assigned to 6 and 7 groups in the first (I) and the second sets (II), respectively. All groups, except group I, was administered a single intraperitoneal (i.p) injection of STZ (65 mg/kg b.w.). Group I received saline and served as healthy control. The two phases consisted of studies using various decreasing dosages to find the ideal dosage of the proposed drug combination (Fig. 5). Set I was carried out as a preliminary dosage screening study with 2–6 rats, while Set II was a validation (*n* = 3) of the best dosage obtained from Set I while testing other decreasing dosages. All analyses were performed on distinct samples.

### Biochemical assays

#### Hypoglycemic effect

Blood collected by retro-orbital puncture was centrifuged to separate the serum. Serum glucose, insulin, and HbA1c levels were measured to determine the hypoglycemic effect of the drug combination. Serum glucose level was measured using a glucose enzymatic kit from Agappe Diagnostics, Kerala, India, per the manufacturer’s instructions. In brief, the GOD-PAP method, an enzymatic colorimetric assay, was used where 10 µl of sample/glucose standard (100 mg/dl) is mixed with 1000 µl of a glucose reagent containing Tris buffer (pH 7.4), phenol, glucose oxidase and 4-aminophenazone for 10 min at 37 °C and the absorbance was measured against a reagent blank at 540 nm. Serum insulin was measured using Mercodia Rat Insulin ELISA Kit and Sweden/RayBio rat insulin ELISA kit (Item: ELR-Insulin) as per the manufacturer’s instructions. Both assay kits are direct sandwich ELISA-based assays where insulin in the sample interacts with peroxidase-conjugated monoclonal antibodies directed against its antigenic determinants and anti-insulin antibodies that the wells of the assay plate are coated with. A washing step removes any excess unbound antibodies, followed by which the insulin concentration is spectrophotometrically measured by incubating it with 3,3’,5,5’-tetramethylbenzidine (TMB) at 450 nm. In brief, 100 µl of each standard and sample are incubated for 2.5 h at room temperature with gentle shaking. The solution is discarded and washed with 1X wash solution (300 µl) four times. 100 µl of 1X biotinylated antibody is then added to each well and incubated at room temperature with gentle shaking. The solution is discarded and washed as previously described, followed by adding 100 µl of streptavidin solution and incubating for 45 min at the same conditions. The solution is then discarded, and the wash step is repeated. 100 µl of TMB one-step substrate reagent is added to each well and incubated for 30 min at room temperature with gentle shaking in the dark. 50 µl of stop solution is then added to each well and read at 540 nm.

Serum HbA1c was also measured using an ELISA-based kit from MyBioSource Inc. (Item no. MBS2880660) as per the manufacturer’s instructions. In brief, 100 µl of standard, blank, and sample were added per well and incubated for 2 h at 37 °C. The liquid was then removed, and 100 µl of anti-HbA1c antibody working solution was added and incubated for 1 h at 37 °C. Each well was aspirated and washed thrice with wash buffer (400 µl). 100 µl of avidin-conjugated Horseradish Peroxidase was then added and incubated for 1 h at 37 °C followed by a washing step. 90 µl of the TMB substrate solution was added and incubated for 15–30 min at 37 °C away from light. Finally, 50 µl of the stop solution was added, and absorbance was read at 450 nm.

#### Lipid Profile

Parameters for the lipid profile - total cholesterol (TC), triglycerides (TG), and high-density lipoprotein (HDL) were determined using enzymatic assay kits obtained from Agappe diagnostic, Kerala, India, as per the manufacturer’s instructions. In brief, the CHOD-PAP method was used to measure TC against a cholesterol standard where 10 µl sample/standard was allowed to react with 1000 µl of a cholesterol reagent containing Pipes buffer, Phenol, Sodium Cholate, 4 -Aminoantipyrine, Cholesterol Esterase, Cholesterol Oxidase, and Peroxidase for 5 min at 37 °C and the concentration was colorimetrically measured at an absorbance of primary wavelength 510 nm and secondary wavelength of 630 nm. Similarly, TG was measured using the GPO-TOPS method against a TG standard by allowing the 10 µl samples/standard to react with 1000 µl TG reagent containing the Pipes–buffer, TOPS, Potassium ferrocynate, Magnesium Salt, 4-Aminoantipyrine, ATP, Lipoprotein Lipase, Glycerol Kinase, Glycerol–3-phosphate oxidase and Peroxidase for 5 min at 37 °C. The absorbance was then measured at a primary wavelength of 546 nm and a secondary wavelength of 630 nm. Further, HDL was measured using a selective inhibition method where the reaction of HLD products with 270 µl of a reagent containing N-Ethyl-N-(3-methylphenyl)-N’succinylethyenediame for 5 min at 37 °C, followed by 90 µl of a reagent containing Cholesterol Oxidase and 4-Aminoantipyrin (4-AA) for 5 min at 37 °C and spectrophotometrically measured at an absorbance of 600 nm and 700 nm for the calibrator and samples. The LDL was calculated using Friedewald’s formula: LDL = TC – (HDL + TG/5).

#### Antioxidant effect

The serum nitrite level (indication of NO release) was determined using the Griess reagent spectrophotometrically. 100 µl of Griess Reagent was added to 300 µl of the nitrite-containing samples and 2.6 ml of deionized water and incubated for 30 min at room temperature. A reference sample was prepared by mixing 100 µl of Griess Reagent and 2.9 ml of deionized water. The absorbance of the sample was measured at 548 nm relative to the reference sample. A standard curve of nitrite concentrations was used to obtain sample nitrite concentrations. Thiobarbituric acid reactive substances (TBARS) assay kit from Cayman chemical company (item no. 10009055) was used for serum measurement of TBARS. TBARS are released as a by-product of lipid peroxidation and serves as an indicator of oxidative stress in the system. Briefly, vials with 100 µl of sample/standard were mixed with 100 µl SDS followed by the addition of 4 ml of color reagent containing Thiobarbituric acid (TBA), TBA acetic acid, and TBA Sodium Hydroxide at 90–100 °C and placed in boiling water for one hour. The vials were then immediately placed in an ice bath for 10 min to stop the reaction. The vials were centrifuged for 10 min at 16,000 x g at 4 °C, and absorbance was measured colorimetrically at 530–540 nm.

Detailed protocols of all assays can be accessed from the vendors’ websites using the kit names and catalog numbers mentioned.

### Histopathology of pancreas

After 28 days, small sections of pancreatic tissue from sacrificed rats were fixed in 10% neutral buffered formalin overnight, and the embedded tissue was processed by dehydrating using ascending grades of 50%, 70%, and absolute alcohol, clearing using two changes of xylene followed by wax impregnation and paraffin embedding. Sections were then made using a microtome and stained with hematoxylin-eosin. In brief, the sections were dewaxed using xylene twice for 5 min each, followed by hydration using two changes of 100% alcohol (3 min each), 95% alcohol (3 min), 80% alcohol (3 min), 70% alcohol (3 min), 50% alcohol (3 min) and rinsing under running tap water for 3 min. The sections were then stained with Hematoxylin for 10 min, followed by rinsing under tap water for 3 min and Eosin staining for 5 min. The tissues were then further passed through successive changes in 95% alcohol twice (3 min), 100% alcohol twice (3 min each), and xylene twice (5 min each) to prevent drying and mounted using DPX and a cover slip and examined under a microscope. The percentage of alpha and beta-pancreatic cells were calculated using the point-counting method based on morphology.

### Statistical analysis

Statistical significance was calculated using ANOVA and Dunnett’s test. All results were expressed as Mean±S.E.M along with their respective *p*-values. Statistical analyses were carried out using GraphPad Prism 9.5.0.

### Reporting summary

Further information on research design is available in the Nature Research Reporting Summary linked to this article.

## Supplementary information

Supplementary Information

Reporting Summary

Dataset 1

Dataset 2

## Data Availability

The publicly available transcriptomic datasets used in the study are accessible at Gene Expression Omnibus using the GSEIDs- GSE18732, GSE13760, GSE20966, GSE23343, and GSE25724. All other data generated and analyzed in the study are presented within the article and its associated supplementary information.

## Code availability

Transcriptomic analysis was carried out using publicly accessible packages, as cited in the methods section, while network-related codes can be obtained from previously published literature from the laboratory, as cited in the methods section.

## References

[1] Mathers CD, Loncar D (2006). Projections of global mortality and burden of disease from 2002 to 2030. PLoS Med..

[2] Rena G, Hardie DG, Pearson ER (2017). The mechanisms of action of metformin. Diabetologia.

[3] Garber AJ (2020). Consensus Statement by the American Association of Clinical Endocrinologists and American College of Endocrinology on the Comprehensive Type 2 Diabetes Management Algorithm – 2020 Executive Summary. Endocr. Pract..

[4] Kashihara N, Haruna Y, K. Kondeti V, S. Kanwar Y (2010). Oxidative stress in diabetic nephropathy. Curr. Med Chem..

[5] Chang YC, Wu WC (2013). Dyslipidemia and diabetic retinopathy. Rev. Diabet. Stud..

[6] Hayat SA, Patel B, Khattar RS, Malik RA (2004). Diabetic cardiomyopathy: Mechanisms, diagnosis and treatment. Clin. Sci. (Lond.).

[7] Rosario RF, Prabhakar S (2006). Lipids and diabetic nephropathy. Curr. Diab. Rep..

[8] Demirbilek H, Galcheva S, Vuralli D, Al-Khawaga S, Hussain K (2019). Ion Transporters, Channelopathies, and Glucose Disorders. Int. J. Mol. Sci..

[9] Ighodaro OM (2018). Molecular pathways associated with oxidative stress in diabetes mellitus. Biomed. Pharmacother..

[10] Perego C (2019). Cholesterol metabolism, pancreatic β-cell function and diabetes. Biochimica et. Biophysica Acta (BBA) - Mol. Basis Dis..

[11] Oh TJ, Shin JY, Kang GH, Park KS, Cho YM (2013). Effect of the combination of metformin and fenofibrate on glucose homeostasis in diabetic Goto-Kakizaki rats. Exp. Mol. Med..

[12] Culafic M (2020). Pentoxifylline with metformin treatment improves biochemical parameters in patients with nonalcoholic steatohepatitis. J. Med Biochem..

[13] Khalaf HM (2019). Allopurinol potentiates the hepatoprotective effect of metformin and vitamin E in fructose-induced fatty liver in rats. Clin. Exp. Hepatol..

[14] De Silva SR, Betteridge DJ, Shawe JE, Cudworth AG, Alberti KG (1989). Metformin and clofibrate in maturity onset diabetes mellitus: advantages of combined treatment. Diabete Metab..

[15] Wishart DS (2006). DrugBank: A comprehensive resource for in silico drug discovery and exploration. Nucleic Acids Res..

[16] Reed MJ (1999). Effect of masoprocol on carbohydrate and lipid metabolism in a rat model of Type II diabetes. Diabetologia.

[17] Saini AK, Kumar H.S A, Sharma SS (2007). Preventive and curative effect of edaravone on nerve functions and oxidative stress in experimental diabetic neuropathy. Eur. J. Pharm..

[18] Gorogawa SI (2002). Probucol preserves pancreatic beta-cell function through reduction of oxidative stress in type 2 diabetes. Diabetes Res Clin. Pr..

[19] Liu HW, Luo Y, Zhou YF, Chen ZP (2020). Probucol prevents diabetes-induced retinal neuronal degeneration through upregulating Nrf2. Biomed. Res Int.

[20] Plomgaard P (2005). Tumor necrosis factor-α induces skeletal muscle insulin resistance in healthy human subjects via inhibition of Akt substrate 160 phosphorylation. Diabetes.

[21] Stumvoll M, Goldstein BJ, van Haeften TW (2005). Type 2 diabetes: Principles of pathogenesis and therapy. Lancet.

[22] Aldana M, Cluzel P (2003). A natural class of robust networks. Proc. Natl. Acad. Sci..

[23] Thieffry D (2007). Dynamical roles of biological regulatory circuits. Brief. Bioinform..

[24] Tanous, D., Hime, N. & Stocker, R. Redox report communications in free radical research anti-atherosclerotic and anti-diabetic properties of probucol and related compounds. 10.1179/135100008X259196 (2013). 10.1179/135100008X259196 18339247

[25] Kaul N (1996). Probucol treatment reverses antioxidant and functional deficit in diabetic cardiomyopathy. Mol. Cell Biochem..

[26] Endo K (2006). Probucol delays progression of diabetic nephropathy. Diabetes Res Clin. Pr..

[27] Aiman U, Najmi A, Khan RA (2014). Statin induced diabetes and its clinical implications. J. Pharm. Pharmacother..

[28] Yamashita S, Matsuzawa Y (2009). Where are we with probucol: A new life for an old drug?. Atherosclerosis.

[29] Yamashita S, Masuda D, Matsuzawa Y (2021). New horizons for probucol, an old, mysterious drug. J. Atheroscler. Thromb..

[30] Lam V (2022). Efficacy of probucol on cognitive function in Alzheimer’s disease: Study protocol for a double-blind, placebo-controlled, randomised phase II trial (PIA study). BMJ Open.

[31] Matsuhashi H (1989). Probucol-induced QT prolongation and torsades de pointes. Jpn J. Med..

[32] Browne KF (1984). Prolongation of the QT interval induced by probucol: Demonstration of a method for determining QT interval change induced by a drug. Am. Heart J..

[33] Ritchie ME (2015). Limma powers differential expression analyses for RNA-sequencing and microarray studies. Nucleic Acids Res..

[34] Sambarey A (2017). Meta-analysis of host response networks identifies a common core in tuberculosis. npj Syst. Biol. Appl..

[35] Sambaturu N, Pusadkar V, Hannenhalli S, Chandra N (2021). PathExt: A general framework for path-based mining of omics-integrated biological networks. Bioinformatics.

[36] Wu G, Feng X, Stein L (2010). A human functional protein interaction network and its application to cancer data analysis. Genome Biol..

[37] Szklarczyk D (2016). STITCH 5: Augmenting protein-chemical interaction networks with tissue and affinity data. Nucleic Acids Res..

[38] Chen EY (2013). Enrichr: Interactive and collaborative HTML5 gene list enrichment analysis tool. BMC Bioinforma..

