# Supplementary material for: Identification of Probucol as a candidate for combination therapy with Metformin for Type 2 diabetes
Source: NPJ Syst Biol Appl. 2023 May 23;9:18. doi: 10.1038/s41540-023-00275-8 (PMC10206066; doi:10.1038/s41540-023-00275-8)
Supplement: Supplementary file 1 — Supplementary Information [file 41540_2023_275_MOESM1_ESM.pdf]

Supplementary files

Supplementary Table 1. Biochemical parameters studied in Study 1. Data are presented as Mean ± S.E.M. * Indicates a statistically significant difference compared to the corresponding group. [a] significantly different from normal control rats. [b] significantly different from untreated diabetic rats (* p-value < 0.05, ** p < 0.01, *** p-value < 0.001, **** p-value < 0.0001; all p-values were calculated using ANOVA followed by Dunnett's test).

Condition	Lipid profile (mg/dl)				Glucose (mg/dl)	Serum HbA1c (ng/ml)	Serum insulin (U/ml)	Serum nitric oxide (µmol/l)
	LDL	HDL	Total cholesterol	Triglycerides				
Normal Control	44.21±2.35	51.51±2.90	107.0±6.87	206.0±12.83	79.37±2.36	12.81±0.60	24.64±0.74	8.97±0.06
Diabetic Control	105.7±6.15****a	29.32±0.35 ****a	180.0±3.93 ***a	332.6±7.48 ***a	312±27.28 ***a	28.66±0.24 ****a	12.11±0.33 ****a	18.73±1.05 ****a
Metformin (5)	64.76±6.53***b	40.66±1.14 ****b	128.9±12.93 *b	282.2±18.36	121.5±5.92 ***b	20.46±0.20 ****b	17.89±0.37 ****b	13.21±0.70 ***b
M+P (5: 5)	60.23±5.65***b	36.12±0.71 ****b	126.8±10.87 *b	281.9±38.11	135.9±4.18 ***b	24.24±0.75**b	15.89±0.66 ***b	10.33±0.78 ****b
M+P (5: 1)	57.24±6.88***b	37.57±2.11 ****b	104.7±1.19 **b	311.4±38.21	104.3±1.18 ***b	18.67±0.32 ****b	18.95±0.41 ****b	10.89±1.37 ****b
M+P (5: 0.5)	53.77±4.08***b	39.04±0.81 ****b	153.4±36.50	206.7±1.74**b	91.61±3.52 ***b	15.271±0.05 ****b	21.84±0.26 ****b	9.95±0.78 ****b

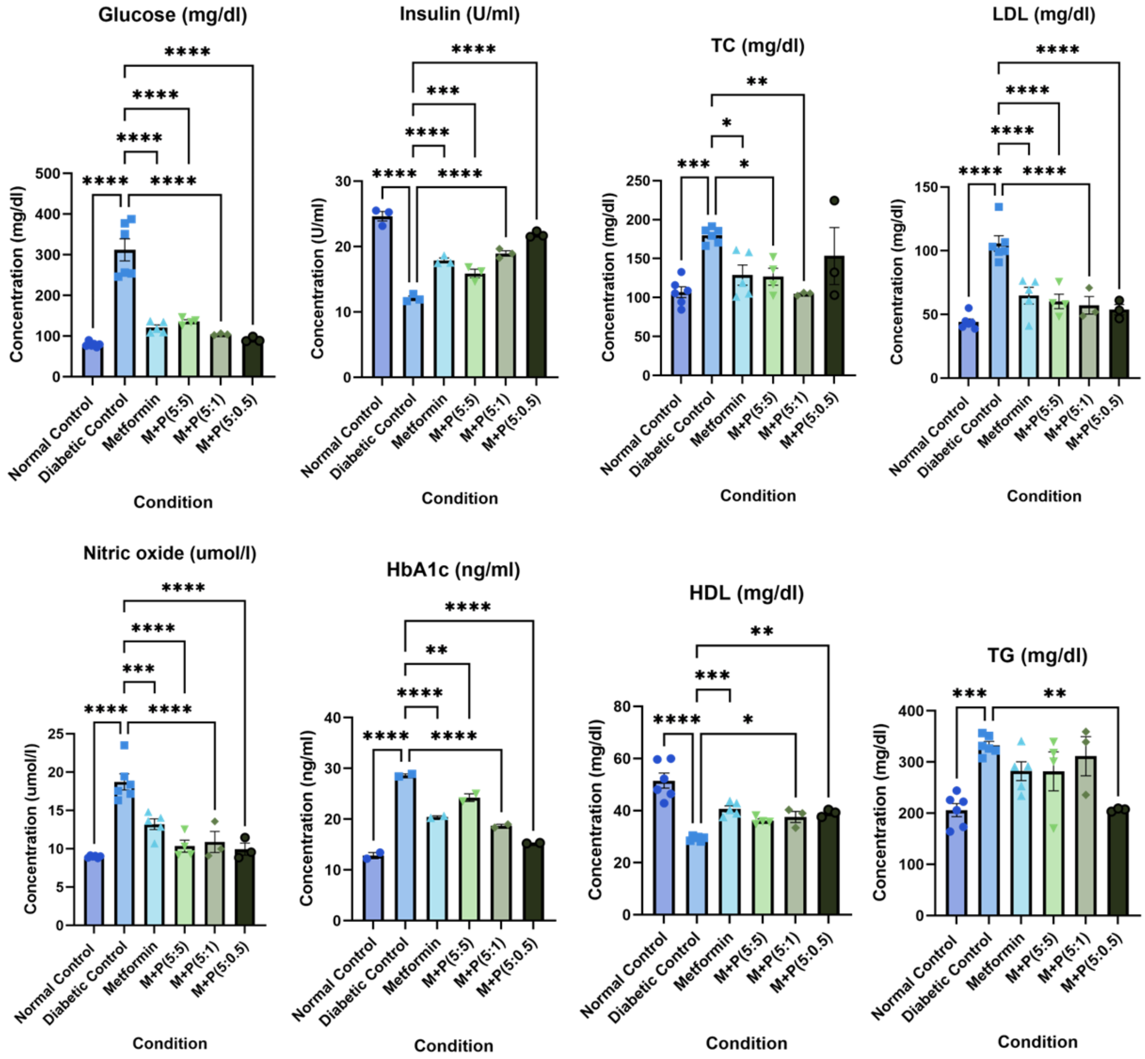

Supplementary Figure 1. Graphical representation of all the biochemical parameters measured in Set I (* p-value < 0.05, ** p < 0.01, *** p-value < 0.001, **** p-value < 0.0001; all p-values were calculated using ANOVA followed by Dunnett's test). Mean with S.E.M are plotted. M+P (5:0.5) shows concentrations closest to the normal healthy control for all parameters. It is hence chosen as the ideal dosage concentration for the drug combination. Some of the rats died before 28 days when the measurements were taken and thus were excluded from the study. Insulin and HbA1c were measured in 3 and 2 rats, respectively, just to validate hyperglycemic changes after glucose measurement. Thus, all rats were not used.

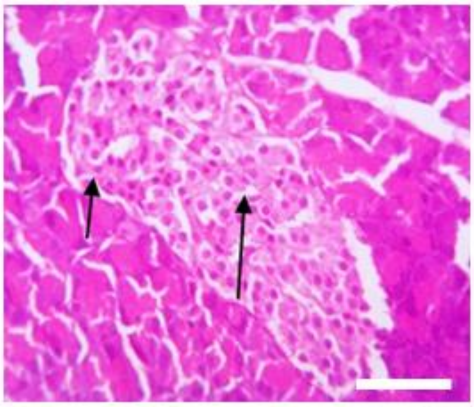

a. Normal (Beta cells – 70%, Alpha cells – 25%)

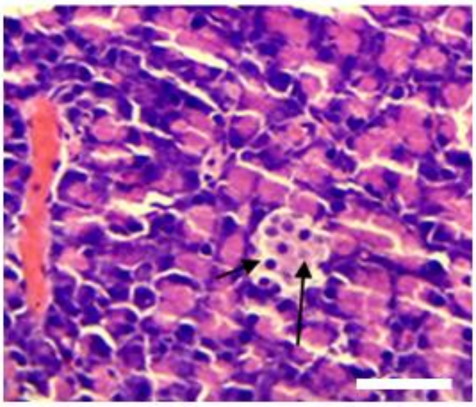

b. Positive (Beta cells – 25%, Alpha cells – 70%)

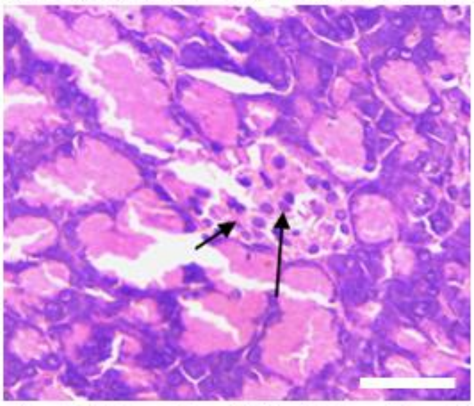

c. Standard (Beta cells – 55%, Alpha cells – 40%)

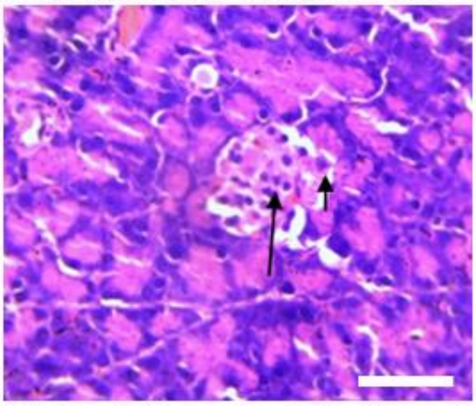

d. M+P Dose I (Beta cells – 40%, Alpha cells – 55%)

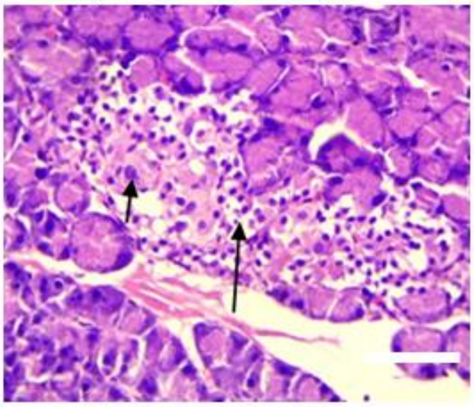

e. M+P Dose II (Beta cells – 65%, Alpha cells – 30%)

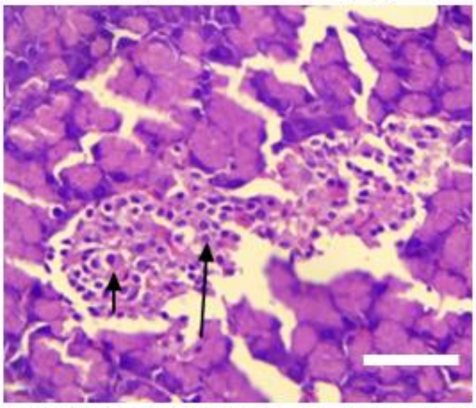

f. M+P Dose III (Beta cells – 70%, Alpha cells – 25%)

Supplementary Figure 2. Histopathological analysis of pancreatic tissue from NAD- STZ induced diabetic model (400x; scale bars = 25 µm). (a) Normal control (Group I, Set I), (b) Diabetic control (Group II, Set I), (c) Metformin treated (standard) (Group III, Set I), (d) Dose I - M+P (5:5) (Group IV, Set I), (e) Dose II - M+P (5:1) (Group V, Set I), (f) Dose III - M+P (5:0.5) (Group VI, Set I). The long arrows represent beta cells, while the short arrows represent alpha cells. M+P (5:0.5) restores normal tissue conditions.
