# Supplementary material for: Identification of Probucol as a candidate for combination therapy with Metformin for Type 2 diabetes
Source: NPJ Syst Biol Appl. 2023 May 23;9:18. doi: 10.1038/s41540-023-00275-8 (PMC10206066; doi:10.1038/s41540-023-00275-8)
Supplement: Supplementary file 2 — Reporting Summary [file 41540_2023_275_MOESM2_ESM.pdf]

nature portfolio

Corresponding author(s): Nagasuma Chandra

Last updated by author(s): Jan 9, 2023

# Reporting Summary

Nature Portfolio wishes to improve the reproducibility of the work that we publish. This form provides structure for consistency and transparency in reporting. For further information on Nature Portfolio policies, see our Editorial Policies and the Editorial Policy Checklist.

## Statistics

For all statistical analyses, confirm that the following items are present in the figure legend, table legend, main text, or Methods section.

| n/a | Confirmed | |
|---|---|---|
| ☐ | ☒ | The exact sample size (*n*) for each experimental group/condition, given as a discrete number and unit of measurement |
| ☐ | ☒ | A statement on whether measurements were taken from distinct samples or whether the same sample was measured repeatedly |
| ☐ | ☒ | The statistical test(s) used AND whether they are one- or two-sided<br>*Only common tests should be described solely by name; describe more complex techniques in the Methods section.* |
| ☐ | ☒ | A description of all covariates tested |
| ☐ | ☒ | A description of any assumptions or corrections, such as tests of normality and adjustment for multiple comparisons |
| ☐ | ☒ | A full description of the statistical parameters including central tendency (e.g. means) or other basic estimates (e.g. regression coefficient) AND variation (e.g. standard deviation) or associated estimates of uncertainty (e.g. confidence intervals) |
| ☐ | ☒ | For null hypothesis testing, the test statistic (e.g. *F*, *t*, *r*) with confidence intervals, effect sizes, degrees of freedom and *P* value noted<br>*Give P values as exact values whenever suitable.* |
| ☒ | ☐ | For Bayesian analysis, information on the choice of priors and Markov chain Monte Carlo settings |
| ☒ | ☐ | For hierarchical and complex designs, identification of the appropriate level for tests and full reporting of outcomes |
| ☒ | ☐ | Estimates of effect sizes (e.g. Cohen's *d*, Pearson's *r*), indicating how they were calculated |

*Our web collection on statistics for biologists contains articles on many of the points above.*

## Software and code

Policy information about availability of computer code

| | |
|---|---|
| Data collection | Publicly available transcriptomic data (GSE18732, GSE13760, GSE20966, GSE23343, and GSE25724) collected from NCBI's Gene Expression Omnibus was used in the study |
| Data analysis | Robust Multi-array Average (RMA) method and Limma package in R 3.4.1 were used for differential expression analysis. An in-house network mining approach was used to shortlist drugs. A general version of the code used can be accessed at https://github.com/NarmadaSambaturu/PathExt. DrugBank was used to obtain the list of drugs. STITCH database was used to obtain drug targets. All networks were visualized using Cytoscape 3.8.2. Functional enrichment for various gene sets and subnetworks was performed using the EnrichR server and the ReactomeFIPlugIn in Cytoscape. |

For manuscripts utilizing custom algorithms or software that are central to the research but not yet described in published literature, software must be made available to editors and reviewers. We strongly encourage code deposition in a community repository (e.g. GitHub). See the Nature Portfolio guidelines for submitting code & software for further information.

## Data

Policy information about availability of data

All manuscripts must include a data availability statement. This statement should provide the following information, where applicable:

- Accession codes, unique identifiers, or web links for publicly available datasets
- A description of any restrictions on data availability
- For clinical datasets or third party data, please ensure that the statement adheres to our policy

The publicly available transcriptomic datasets used in the study are accessible at Gene Expression Omnibus using the GSEIDs- GSE18732, GSE13760, GSE20966, GSE23343, and GSE25724. All other data generated and analyzed in the study are presented within the article and its associated supplementary information.

## Human research participants

Policy information about studies involving human research participants and Sex and Gender in Research.

| | |
|---|---|
| Reporting on sex and gender | Not applicable |
| Population characteristics | Not applicable |
| Recruitment | Not applicable |
| Ethics oversight | Not applicable |

Note that full information on the approval of the study protocol must also be provided in the manuscript.

# Field-specific reporting

Please select the one below that is the best fit for your research. If you are not sure, read the appropriate sections before making your selection.

☒ Life sciences ☐ Behavioural & social sciences ☐ Ecological, evolutionary & environmental sciences

For a reference copy of the document with all sections, see nature.com/documents/nr-reporting-summary-flat.pdf

# Life sciences study design

All studies must disclose on these points even when the disclosure is negative.

| | |
|---|---|
| Sample size | Study 1 included 2-6 animals while Study 2 included 3 animals in each group. |
| Data exclusions | Animals that died within 28 days of treatment were excluded from the study. |
| Replication | Multiple animals were used in each group to account for variability. All raw data for individual rats as well as statistical information has been provided for ease of replication. |
| Randomization | All animals were assigned to different groups randomly. |
| Blinding | Not applicable. |

# Reporting for specific materials, systems and methods

We require information from authors about some types of materials, experimental systems and methods used in many studies. Here, indicate whether each material, system or method listed is relevant to your study. If you are not sure if a list item applies to your research, read the appropriate section before selecting a response.

## Materials & experimental systems

| n/a | Involved in the study |
| --- | --- |
| ☒ | ☐ Antibodies |
| ☒ | ☐ Eukaryotic cell lines |
| ☒ | ☐ Palaeontology and archaeology |
| ☐ | ☒ Animals and other organisms |
| ☒ | ☐ Clinical data |
| ☒ | ☐ Dual use research of concern |

## Methods

| n/a | Involved in the study |
| --- | --- |
| ☒ | ☐ ChIP-seq |
| ☒ | ☐ Flow cytometry |
| ☒ | ☐ MRI-based neuroimaging |

# Animals and other research organisms

Policy information about studies involving animals; ARRIVE guidelines recommended for reporting animal research, and Sex and Gender in Research

| | |
| --- | --- |
| Laboratory animals | Healthy Wistar albino rats weighing 150–180g and 180-230g were selected for acute oral toxicity study and anti-diabetic activity, respectively. |
| Wild animals | The study did not involve wild animals. |
| Reporting on sex | Female rats were used for oral toxicity studies as the OECD has established guidelines recommending acute oral toxicity testing to be done only in female rats when there is no indication that males are more sensitive to the compound being tested (OECD, 1998). All other analyses were done using rats of either sex. |
| Field-collected samples | The animals were procured from the Drug control department, Bengaluru, India and housed in polypropylene cages maintained under standard hygienic laboratory conditions at a temperature of 23±2°C, relative humidity of 55±5%, and 12-hour light and dark cycles with free access ad libitum to a standard pellet diet and water. |
| Ethics oversight | The experimental protocol was approved by KLE University's College of Pharmacy, Bengaluru's Institutional Animal Ethics Committee (Ref. no: 01/PA/2016) |

Note that full information on the approval of the study protocol must also be provided in the manuscript.
